# Effects of *Cannabis sativa* L. Leaves on *Opisthorchis viverrini* Metacercariae in Infected *Barbonymus gonionotus*

**DOI:** 10.1155/japr/6233585

**Published:** 2025-11-11

**Authors:** Naiyana Senasri, Nattiya Chumnanka, Patcharawalai Sriyasak, Supannee Suwanpakdee, Kosit Sriputhorn, Chaweng Sarnklong, Sutee Wongmaneeprateep

**Affiliations:** ^1^Department of Fisheries, Faculty of Natural Resources, Rajamangala University of Technology Isan Sakon Nakhon Campus, Sakon Nakhon, Thailand; ^2^Department of Animal Science, Faculty of Natural Resources, Rajamangala University of Technology Isan Sakon Nakhon Campus, Sakon Nakhon, Thailand; ^3^Department of Fisheries, Faculty of Agriculture, Khon Kaen University, Khon Kaen, Thailand

## Abstract

This study is aimed at evaluating the effects of dietary *Cannabis sativa* L. leaf supplementation on superoxide dismutase (SOD) levels and the prevention of liver fluke (*Opisthorchis viverrini*) metacercaria infection in *Barbonymus gonionotus*. The experiment included five treatment groups, with varying concentrations of *C. sativa* leaves (0.0%, 0.5%, 1.0%, 1.5%, and 2.0%) in the experimental feed. Six hundred parasite-free *B. gonionotus* (50 days old) were infected with 50 cercariae each. After 24 h, they were fed the experimental feed for 0 (control group), 7, 14, or 21 days. The infection rate, intensity of *O. viverrini* metacercaria, survival rates, immunoglobulin M (IgM) levels, lysozyme activity, and SOD levels in *B. gonionotus* were investigated. The results showed that cannabis leaves effectively prevented *O. viverrini* infection. Fish fed with higher doses of cannabis leaf diets had a decreased infection rate and intensity of *O. viverrini* metacercariae and higher survival rates. Conversely, there was an increase in SOD, lysozyme activity, and IgM levels. Moreover, after the fish were fed 2.0% cannabis leaves for 14 and 21 days, no *O. viverrini* metacercariae were degenerated. The highest SOD levels were exhibited by fish fed 2.0% cannabis leaves for 14 days (1497.96 U/g FW), and the metacercariae were inactive and degenerated. In summary, dietary supplementation of cannabis leaves can be used as a preventive measure against liver fluke infection in *B. gonionotus*.

## 1. Introduction

Opisthorchiasis is a disease caused by trematode infection in fish, specifically members of the Opisthorchiidae family, including *Opisthorchis viverrini*, *Opisthorchis felineus*, and *Clonorchis sinensis* [1]. *O. viverrini* infection in particular poses a significant public health issue along the Mekong River in Thailand, Vietnam, Laos, Cambodia, China, and Myanmar [2, 3]. It is estimated that approximately 10 million people in these countries are infected with the *O. viverrini* (liver fluke), with over 6 million cases in Thailand, mainly in the northeastern region. Meanwhile, it is estimated that there are over 2 million infected individuals in Laos [4, 5]. Chronic *O. viverrini* infection is associated with the development of liver disease and cholangiocarcinoma (CCA) [6]. In Thailand, the annual cost of medical treatment and wages lost due to *O. viverrini* infection is estimated to be around $120 million [7]. Controlling the liver fluke disease is difficult because of its complex life cycle, including hosts, environmental conditions, and the intricate process of disease transmission [1, 8]. Consuming undercooked food, especially raw fish salad (Koi pla) popular dish in the northeastern region of Thailand, is a risk factor for contracting the *O. viverrini* infection. This is a significant factor contributing to the spread of *O. viverrini* infection [9, 10]. Cyprinid fish that are consumed and infected with *O. viverrini* are mostly found in several species in the upper northeastern region, such as *Mystacoleucus obtusirostris* (52.94%), *Osteochilus melanopleura* (43.47%), *Henicorhynchus siamensis* (13.21%), *Cyclocheilichthys repasson* (6.09%), and *Osteochilus hasselti* (5.06%) [11]. Therefore, preventing *O. viverrini* infection in fish is important for controlling the spread of liver fluke disease in humans.

Cannabis, *Cannabis sativa* L., possesses a rich historical background of human utilization, with indications of its use dating back thousands of years. The functions of cannabis have evolved across different periods and regions, encompassing activities such as fiber production and therapeutic applications [12, 13]. It is a plant that is legalized for medical or recreational use in many countries. Currently, the Thai government allows the use of cannabis for medical purposes in compliance with existing legislation. Cannabis contains various cannabinoids, with the most predominant active compounds being cannabidiol (CBD) and tetrahydrocannabinol (THC) [14]. The total cannabinoids quantified in leaves were between 1.10% and 2.10% [15], which agreed with the previously reported amounts (1%–2% and 1.40%–1.75%) [16, 17]. Cannabis supplementation does not significantly affect the body composition of tilapia but reduces growth rate by increasing metabolic rate [18]. While THC exhibits psychoactive properties, CBD is a pharmacologically active compound with significant therapeutic potential for various diseases and possesses anti-inflammatory characteristics [19, 20]. It is anticipated that cannabis extracts may possess therapeutic properties for disease treatment through the suppression of inflammatory molecule production [21]. In addition, it has also been found that CBD may be associated with both specific and nonspecific immune responses [22]. According to the study by Esposito et al. [20], it was found that the bioactive compounds in cannabis leaves exhibited anti-inflammatory effects in RAW 264.7 macrophage cells induced by lipopolysaccharide. Additionally, cannabis tissue as a whole has been shown to possess anti-inflammatory properties. The aim of the study was to investigate the effects of cannabis leaves on liver fluke infection and the immune response in fish.

## 2. Materials and Methods

### 2.1. Effects of Dietary Supplementation With Cannabis Leaves on *O. viverrini* Metacercariae in Infected *B. gonionotus*

#### 2.1.1. Study Design

A completely randomized design (CRD) was used in this experiment, assigning 450 fish to five groups of experimental dietary treatments with three replicates (10 fish each). The cannabis leaves were supplemented at different levels of 0.0%, 0.5%, 1.0%, 1.5%, and 2.0% for *B. gonionotus* to study the effectiveness of cannabis leaves on preventing *O. viverrini* metacercariae infection in fish.

#### 2.1.2. Experimental Parasite-Free Fish

White *B. gonionotus* were bred and reared with commercial powdered feed in a 120 × 150 × 80-cm cement tank to ensure the fish were free from pathogens before conducting the experiments. Tap water was treated to remove chlorine and was aerated throughout the experimental period. When the fish reached 50 days old, they were infected with *O. viverrini* cercariae. Newly shed cercariae were used in this study. Briefly, freshwater snails (*Bithynia siamensis goniomphalos*) were placed individually in plastic cups (diameter = 3 cm and height = 2.5 cm) that contained 10 mL of dechlorinated tap water. The *O. viverrini* cercariae were released from snails into the water by using the cercarial shedding method (induced by exposure to electric light [40 W] for 2–3 h) and counted under a stereomicroscope for use in fish infection. They identified it under a light microscope using the morphological characteristics of a tobacco pipe shaped when briefly hanged head down or laid on the bottom, a pair of eye spots, and a long tail with fins on both lateral sides [23–25]. The cercariae identification was confirmed by polymerase chain reaction (PCR) analysis using species-specific primers, OV-6F (5⁣′-CTG AAT CTC TCG TTT GTT CA-3⁣′) and OV-6R (5⁣′-GTT CCA GGT GAG TCT CTC TA-3⁣′) [26]. The result showed an *O. viverrini*–specific amplified DNA band of 330 bp.

#### 2.1.3. Experimental Feed

*B. gonionotus* were reared and fed the experimental diet supplemented with cannabis leaves at different levels to assess the effectiveness of cannabis leaves on preventing liver fluke infection at the metacercarial stage. The chemical composition of cannabis leaves was analyzed. The nutrient composition of the cannabis leaves was found to be 7.32% protein, 2.67% moisture, 17.33% ash, 19.47% fiber, and 1.38% fat.

#### 2.1.4. Experimental Setup and Data Collection

A group of 1000 50-day-old *B. gonionotus*, each weighing between 0.50 and 0.60 g on average, was chosen randomly and placed in a 30-L glass tank, with 10 fish in each tank (a total of 30 fish per treatment) across 15 tanks (divided into five treatment groups, with three replications). Subsequently, the fish were fed a diet mixed with cannabis leaves for 0, 7, 14, and 21 days, after which they were infected with *O. viverrini* cercariae (50 cercariae per fish). After 24 h of *O. viverrini* cercarial infection, the fish were subjected to cold shock and placed on slides to determine the number of *O. viverrini* metacercariae under a microscope at 40× magnification, and the results were recorded [27]. (*O. viverrini* cercariae was collected from the freshwater snail, *Bithynia siamensis goniomphalos*, which is the primary host responsible for *O. viverrini* transmission in endemic areas.)

#### 2.1.5. The Immune Response

After recording the *O. viverrini* metacercariae in the fish tissue, the fish tissue was collected to investigate the superoxide dismutase (SOD) activity according to the method of Senasri et al. [27]. In each experimental group, the fish were first subjected to bloodletting to remove all blood from the tissues using phosphate-buffered saline (PBS), pH 7.4. The tissues were then finely ground, and 300 mg was weighed. Subsequently, 3–5 mL of 0.1 M Tris/HCL, pH 7.4, containing 0.5% Triton X-100, 5 mM *β*-ME, and 0.1 mg/mL PMSF, were added. The mixture was then centrifuged at 14,000 × *g* for 5 min at 4°C. The supernatant was collected to investigate the level of SOD activity with the Superoxide Dismutase Activity Assay Kit (Colorimetric) (ab65354; Abcam) using ELISA 450 nm (ELISA reader, Infinite F50, TECAN, Switzerland).

Immunoglobulin M (IgM) in serum was determined using a single radial immunodiffusion assay (SRID). A 1% of melted Bio-Rad agarose, standard low *m*_*r*_, with 2% PEG-6000 in 0.01 M PBS, pH 7.2, was prewarmed to 55°C. Rabbit anti–*B. gonionotus* IgM was mixed with agar solution, resulting in a final serum dilution of 1:250. The antiserum–agar mix 0.1 mL/cm was poured onto a GelBond film plate. Serum was applied to 4-mm wells in the gel and incubated in a humid atmosphere for 48 h. The gel was squeezed and washed three times in PBS, dried before staining with Coomassie Blue for 10 min, and subsequently destained for 6 min. The standard curve was derived from four concentrations of purified *B. gonionotus* IgM. Protein concentration of purified IgM was determined by the Bio-Rad assay, using BSA as protein standard. Serum IgM levels were recorded as milligrams per milliliter [28]. The purification of *B. gonionotus* IgM was performed using the Pharmacia FPLC chromatographic system (Pharmacia, Uppsala, Sweden) as described by Havarstein et al. [29]. *B. gonionotus* IgM was used as a standard for the quantification of serum IgM.

Lysozyme activity was determined using a modification of the method described by Chen et al. [30]. A 100 mL of fish serum was added to a 3 mL suspension of *M. lysodeikticus*. The reaction was carried out at 25°C ± 1°C, and an absorbance at 540 nm was measured after 0.5 and 4.5 min. One unit of lysozyme activity was defined as the amount of lysozyme producing a decrease in absorbance of 0.001 in 1 min.

The data were collected to analyze the differences in infection rates of liver fluke in experimental groups by finding the percentage of liver fluke infection rate, determining the intensity of liver fluke infection (metacercaria per fish), and calculating the percentage of survival rate of liver fluke at the metacercaria stage [31].

### 2.2. The Effect of Dietary Supplementation With Cannabis Leaves on Histopathology of *O. viverrini–*Infected Silver Barb Fed Fish

After being infected with *O. viverrini* for 24 h, 10 fish from each treatment were randomly sampled and soaked in 10% formalin for 24 h. The whole fish was decalcified in 0.5 M AlCl_3_·6H_2_O, 2.6 M HCl, and 1.325 M formic acid for 4–5 h. The samples were rinsed with distilled water 3X and placed in 5% Na_2_SO_4_ for 4–5 h. After decalcification, each sample was dehydrated through a graded ethanol series and embedded in paraffin wax. Five-micrometer-thick GMA sections were mounted on normal microscope slides. The 5-mm-thick paraffin sections were mounted with hematoxylin and eosin (H&E) to assess the morphological effects of the different dietary supplementation for silver barb [32].

### 2.3. Statistical Analysis

A one-way analysis of variance (ANOVA) was conducted, and the differences between all experimental treatments were compared using Duncan's new multiple range test (DMRT) at a 95% confidence level. The statistical analysis was performed using IBM SPSS Statistics software Version 22 (IBM Corp., United States).

## 3. Results

### 3.1. The Effect of Dietary Supplementation With Cannabis Leaves on the *O. viverrini* Metacercaria–Infected Fish

The results of the investigation of the prevalence of *O. viverrini* metacercariae in *B. gonionotus* fed a diet supplemented with cannabis leaves at different levels for 0, 7, 14, and 21 days are shown in Table 1. The results showed that increased levels of cannabis leaf supplementation in the feed and longer feeding period were beneficial to *O. viverrini* infection. At 7 days, the infection rates of infected fish fed diet with cannabis leaf supplementation of 0.5%, 1.0%, 1.5%, and 2.0% was 80.00% ± 0.00%, 76.67% ± 5.77%, 73.33% ± 5.77%, and 63.33% ± 5.77%, respectively, which were significantly lower than the control group at 93.33% ± 5.77% (*p* < 0.05). The fish fed a diet with cannabis leaf supplementation of 2.0% for 14 and 21 days were observed to have an infection rate of 0.00%, while the highest infection rate was found in the control group at 93.33% ± 5.77% (*p* < 0.05) compared to all treatments. At 21 days, the fish fed a diet with cannabis leaf supplementation of 0.5%, 1.0%, and 1.5% were observed to have the infection rate at 63.33% ± 5.77%, 46.67% ± 5.77%, and 13.33% ± 5.77%, respectively, which was lower than the control group at 93.33% ± 5.77%.

The results of the investigation of the intensity of *O. viverrini* metacercariae infection in *B. gonionotus* fed a diet with supplementation of cannabis leaves at different levels are shown in Table 2. Fish fed diet supplemented with 0.5%, 1.0%, 1.5%, and 2.0% cannabis leaves for 7 days exhibited infection intensities of 9.54 ± 0.47, 8.25 ± 0.69, 4.15 ± 0.59, and 0.74 ± 0.10 metacercariae per fish, respectively, which were significantly lower than the control group's intensity of 11.13 ± 0.15 metacercariae per fish (*p* < 0.05). The fish fed a diet supplemented with 2.0% cannabis leaves for 14 days demonstrated the lowest intensity of *O. viverrini* metacercariae infection at 0.00 ± 0.00 metacercaria per fish, significantly lower compared to the control group at 10.37 ± 0.86 metacercaria per fish (*p* < 0.05). At 21 days, the highest intensity of *O. viverrini* metacercariae infection was found in the control group, with 11.13 ± 0.77 metacercariae per fish. In contrast, fish fed a diet supplemented with 2.0% cannabis leaves had the lowest intensity of infection, significantly lower than the control group (*p* < 0.05).

Table 3 displays that the survival rates of *O. viverrini* metacercariae in *B. gonionotus* increased with decreasing cannabis leaf supplementation. At 7 days, the fish fed a diet supplemented with 2% cannabis leaves demonstrated the lowest survival rate at 0.46 ± 0.11, compared to the control group (20.20 ± 0.52) (*p* < 0.05). At 14 and 21 days, fish receiving a 2.0% supplementation of cannabis leaves exhibited the lowest *O. viverrini* survival rate at 0.00% ± 0.00%. In the group of 1.5% supplementation at 14 and 21 days, the survival rates were not statistically different (*p* > 0.05) (0.13% ± 0.11% and 0.26% ± 0.11%). However, a statistically significant difference (*p* < 0.05) was observed when comparing these groups to the fish that did not receive any cannabis leaf supplementation, with survival rates of 19.93% ± 0.46% and 19.86% ± 0.30% for the two time periods, respectively.

**Figure 1:**
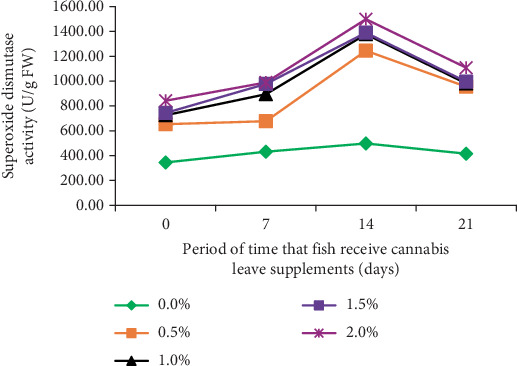
Level of superoxide dismutase activity (SOD) in silver barb fish fed a diet supplemented with cannabis leaves at different levels. Levels of dietary supplementation of cannabis leaves in B. gonionotus were 0.0%, 0.5%, 1.0%, 1.5%, and 2.0%.

The immune response of fish was examined by assessing SOD activity in *B. gonionotus* fed with different levels of cannabis leaf supplementation for varying durations of 0, 7, 14, and 21 days. The results indicated that fish in all experimental groups exhibited elevated immune responses compared to the control group (Figure 1). Specifically, the fish fed a diet supplemented with 2.0% cannabis leaves for 14 days demonstrated the highest SOD value (1497.96 U/g FW), whereas the control group exhibited the lowest value (498.20 U/g FW). Furthermore, the decline in SOD activity after 14 days observed in all groups, including the control group, suggests that the reduction is likely due to physiological adaptation or a time-dependent regulatory mechanism rather than the effect of cannabis leaf supplementation.

**Figure 2:**
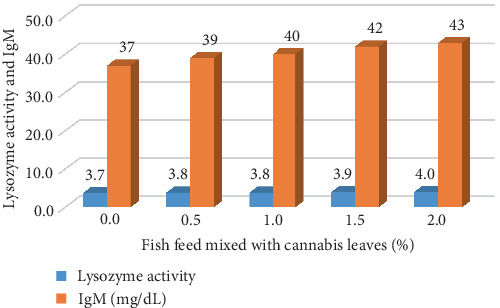
Immunoglobulin M (IgM) levels and lysozyme activities in silver barbs fed with various levels of cannabis leaf supplementation after a period of 4 months.

*B. gonionotus* was raised for 4 months and tested for immunity by measuring the IgM levels and lysozyme activity. The IgM levels and lysozyme activity in fish fed with 2% cannabis leaf supplementation showed the highest values of 43 mg/dL and 4.0, respectively. Meanwhile, the control group of fish that did not receive cannabis leaf supplementation exhibited lower values, with IgM at 37 mg/dL and lysozyme activity at 3.7 (Figure 2).

### 3.2. The Effects of Dietary Supplementation With Cannabis Leaves on Histology of *O. viverrini* Metacercariae–Infected *B. gonionotus*

**Figure 3:**
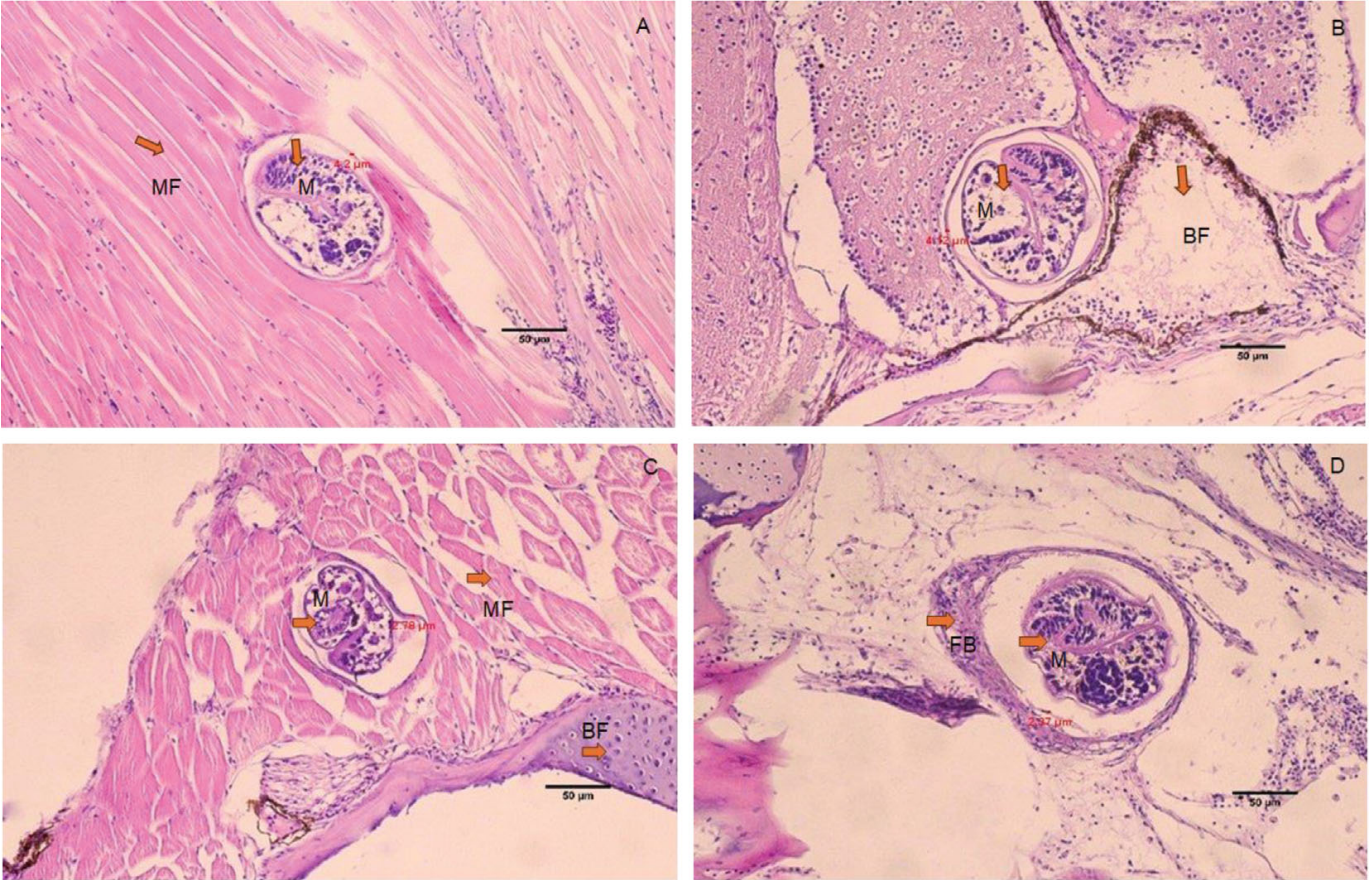
Histology of *O. viverrini* metacercariae–infected *B. gonionotus* fed diet supplemented with 0.0% cannabis leaves at different feeding periods. (A) 0 day (control) (4.20 *μ*m), no fibrous tissue; (B) 7 days (4.12 *μ*m), no fibrous tissue; (C) 14 days (2.78 *μ*m), no fibrous tissue; and (D) 21 days (2.37 *μ*m), fibrous tissue was observed encircling the metacercaria. M, metacercaria; FB, fibrous tissue; MF, striated muscle of fish; BF, bone of fish (H&E, scale bar = 50* μ*m). The thickness of the outer cyst wall of the metacercaria in fish fed a diet containing 0.0% cannabis leaves was measured.

**Figure 4:**
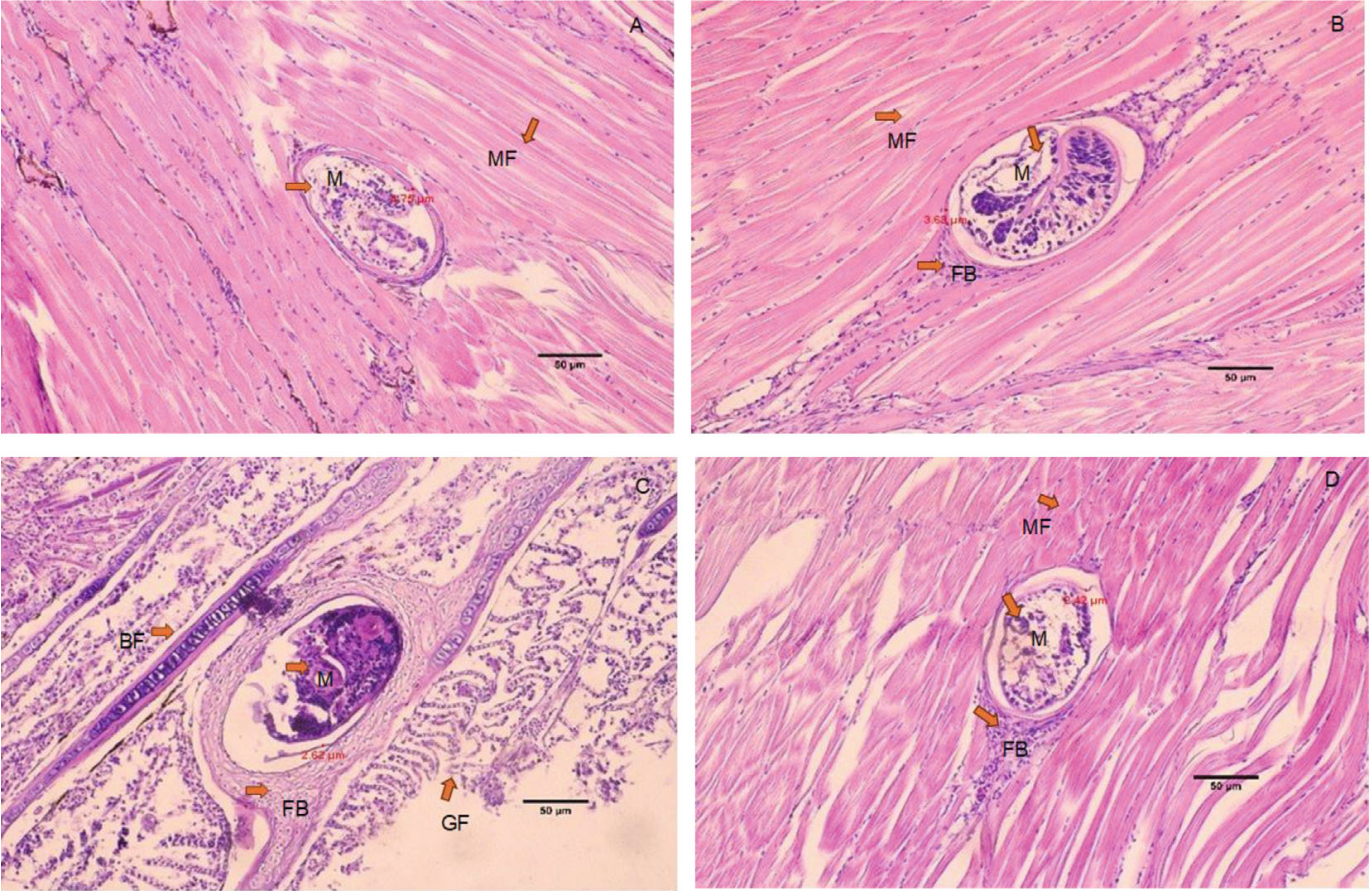
Histology of *O. viverrini* metacercariae–infected *B. gonionotus* fed diet supplemented with 0.5% cannabis leaves at different feeding periods. (A) 0 day (control) (4.75 *μ*m), no fibrous tissue; (B) 7 days (3.63 *μ*m), white blood cells began to surround the metacercaria; (C) 14 days (2.62 *μ*m), white blood cells and fibrous tissue were observed enveloping the cysts in a dense layer, and (D) 21 days (2.42 *μ*m), the metacercaria cysts were encapsulated by fibrous tissue to an extent where the metacercaria underwent desiccation and perished. The thickness of the outer cyst wall of the metacercaria in fish fed a diet containing 0.5% cannabis leaves was measured. M, metacercaria; FB, fibrous tissue; MF, striated muscle of fish; BF, bone of fish; GF, gill of fish (H&E, scale bar = 50* μ*m).

**Figure 5:**
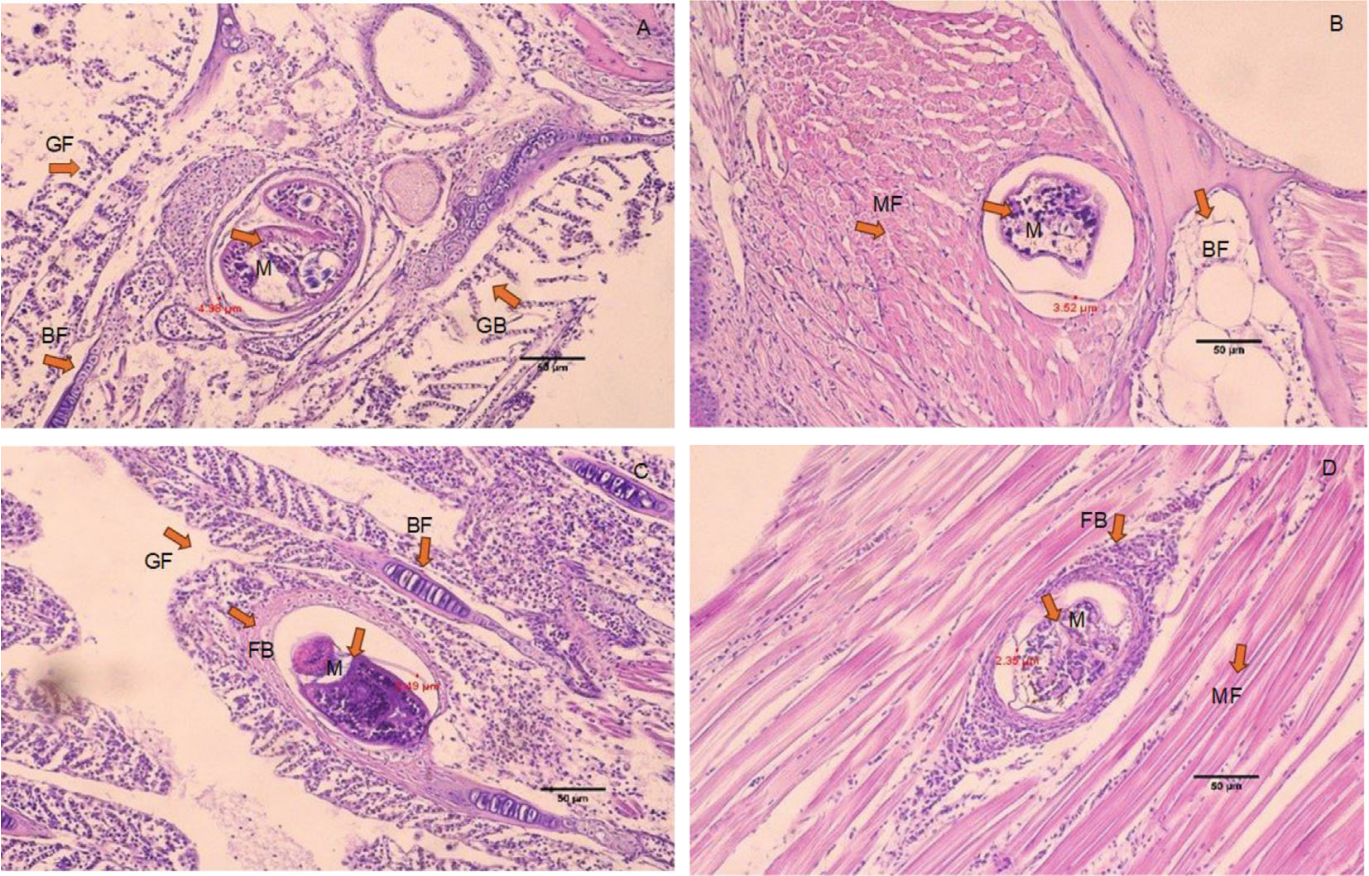
Histology of *O. viverrini* metacercariae–infected *B. gonionotus* fed diet supplemented with 1.5% cannabis leaves at different feeding periods. (A) 0 day (control) (4.98 *μ*m), no fibrous tissue, (B) 7 days (3.52 *μ*m), white blood cells began to surround the metacercaria, (C) 14 days (2.49 *μ*m), white blood cells and fibrous tissue were observed enveloping the cysts in a dense layer, and (D) 21 days (2.35 *μ*m), the metacercaria cysts were encapsulated by fibrous tissue to an extent where the metacercaria underwent desiccation and perished. The thickness of the outer cyst wall of the metacercaria in fish fed a diet containing 1.5% cannabis leaves was measured. M, metacercaria; FB, fibrous tissue; MF, striated muscle of fish; BF, bone of fish; GF, gill of fish (H&E, scale bar = 50* μ*m).

**Figure 6:**
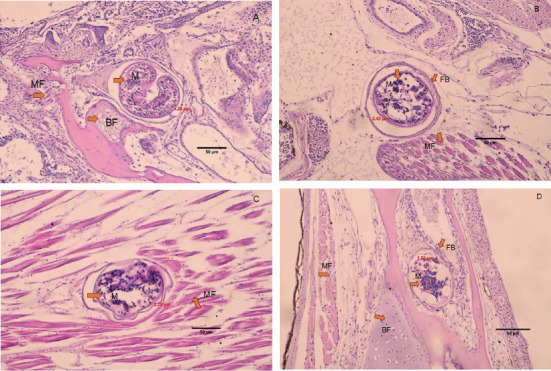
Histology of *O. viverrini* metacercariae–infected *B. gonionotus* fed diet supplemented with 2% cannabis leaves at different feeding periods. (A) 0 day (control) (4.23 *μ*m), (B) 7 days (2.43 *μ*m), white blood cells began to surround the metacercaria, (C) 14 days (2.28 *μ*m), white blood cells and fibrous tissue were observed enveloping the cysts in a dense layer, and (D) 21 days (1.03 *μ*m), the metacercaria cysts were encapsulated by fibrous tissue to an extent where the metacercaria underwent desiccation and perished. The thickness of the outer cyst wall of the metacercaria in fish fed a diet containing 0.5% cannabis leaves was measured. M, metacercaria; FB, fibrous tissue; MF, striated muscle of fish; BF, bone of fish; GF, gill of fish; MS, larvae were degenerated (H&E, scale bar = 50* μ*m).

The histology of fish clearly showed the presence of metacercaria in the control group (without cannabis supplementation), with no fibrous tissue formation surrounding *O. viverrini* metacercariae, only a thin cyst wall produced by worms (Figures 3A, 3B, and 3C). At 21 days, fibrous tissue was observed encircling the metacercaria (Figure 3D). At 7 days, white blood cells in fish fed diets supplemented with 0.5%–2.0% cannabis leaves began to surround the metacercaria, although the parasites remained clearly visible (Figures 4B, 5B, and 6B). Except for the control group (Figures 4A and 5A), in which no fibrous tissue formation was observed surrounding *O. viverrini* metacercariae, only a thin cyst wall produced by the worms was present. Following a 14-day consumption of feed supplemented with cannabis leaves, a substantial accumulation of white blood cells and fibrous tissue was observed enveloping the cysts in a dense layer, rendering the metacercaria imperceptible (Figures 4C, 5C, and 6C). By the end of the 21-day period of ingesting the cannabis leaf–supplemented feed, the metacercaria cysts were encapsulated by fibrous tissue to an extent where the metacercaria underwent desiccation and perished (Figures 4D, 5D, and 6D). However, the fish fed a diet supplemented with 2.0% cannabis leaves at 14 and 21 days showed degeneration of the larvae inside. The cyst wall thickness of metacercariae in fish fed 0%–2% cannabis leaves was measured. The cyst wall of the metacercaria consists of two layers: the inner cyst wall, which is thin and produced by the worm, and the outer cyst wall, which is thick and produced by the fish. In the 0%–1.5% group, only the inner cyst wall was observed. On Day 0 (control), the cyst wall thickness was 4.23 *μ*m (Figure 6A). On Day 7, the cyst wall thickness decreased to 2.43 *μ*m (Figure 6B). On Day 14, the cyst wall thickness was reduced further to 2.28 *μ*m. By Day 21, the cyst wall thickness had decreased to 1.03 *μ*m. These findings indicate that fish fed 2% cannabis leaves for 21 days exhibited the greatest improvement in immunity (Figures 6A, 6B, 6C, and 6D).

## 4. Discussion

The supplementation of cannabis leaves in the silver barb diet has exhibited beneficial effects associated with lowering *O. viverrini* infection. According to the US Food and Drug Administration and other regulatory agencies, terpenoids and phytocannabinoids in food should be maintained at safe levels [33]. This research represents the first investigation to employ cannabis leaves as a supplement in fish feed to reduce the infection of *O. viverrini* metacercariae in *B. gonionotus*. The group of fish that consumed feed supplemented with cannabis leaves exhibited a reduction in *O. viverrini* infection, with the degree of reduction correlating with the concentration of cannabis leaves in the feed. Fish infected with *O. viverrini* metacercariae and provided a diet supplemented with 2.0% cannabis leaves for durations of 14 and 21 days showed degeneration of *O. viverrini* metacercariae. The intensity of infection in this group and the survival rate of metacercaria in fish were significantly reduced. Conversely, fish in the control group that did not receive cannabis leaves in their diet displayed infection. This can potentially be attributed to the presence of CBD in cannabis leaves, a phytocannabinoid known for its diverse pharmacological properties [34], including the ability to resist the bacteria *Staphylococcus aureus* (methicillin-resistant) [34, 35]. Therefore, this indicates that it is possible to effectively prevent infection with *O. viverrini* in fish. This is consistent with the study by Senasri et al. [27], which reported that *B. gonionotus* did not show infection with metacercariae of liver fluke, and displayed low infection density in fish and minimal recovery of metacercariae (0.00%) within 14 days of receiving a diet supplemented with vitamin C. Additionally, throughout the experimental period, the SOD levels were the lowest in the group that did not receive cannabis supplementation, while the fish fed cannabis leaves at a concentration of 2.0% for 14 days demonstrated the highest SOD value of 1497.96 U/g FW. This value was three times greater than that of the control group, aligning with findings from a study by Senasri et al. [27] involving *B. gonionotus* infected with *O. viverrini* metacercaria. Their results indicated that fish provided with a vitamin C supplement at a dosage of 2000 mg/kg for 14 days displayed elevated SOD levels compared to the control group. Similarly, Donthaisong et al. [36] reported that fish infected with *O. viverrini* and treated with prednisolone (an immunosuppressive drug) showed a decrease in SOD levels. This suggests that lower immunity in fish results in decreased SOD levels and higher infection rates. Conversely, if SOD levels are high, the infection rate of liver fluke parasites decreases as well. This study also found that SOD levels tended to decrease when fish were fed a diet supplemented with cannabis leaves for 21 days. The utilization of cannabis as a dietary supplement for immune stimulation has been indicated to bolster the body's innate defense mechanisms against infections and bolster resistance to specific diseases [37]. This approach may lead to a rapid enhancement in immune response time, and memory unit development may not be hindered [38]. Consequently, it is plausible that the natural immune response may gradually strengthen following exposure to an infection, with immunity potentially beginning to rise, as indicated by elevated levels of SOD, within a timeframe of 7–14 days, although SOD levels may decline over time. In addition, fish fed with 2% cannabis leaves were found to have the highest lysozyme activity and IgM values, which shows that cannabis leaves mixed in feed recipes influence the immune system in fish. In a similar study by Soltanian and Fereidouni [39], the highest lysozyme activity was observed in Henna (*Lawsonia inermis*) extract in medium-dose treatment (the extract at 60 and 600 mg kg^−1^ BW). Research by Wang et al. [40] on the effects of CBD on the growth performance of juvenile large yellow croaker (*Larmichthys crocea*) (no infected fish) fed diets found that CBD supplemented in diets with high soybean oil level improved the growth performance and appetite in large yellow croaker. In addition, supplementation with 100 mg/kg CBD in soybean oil diets increased peroxidase activity and total antioxidant capacity compared to the control group and had the highest SOD activity, which was similar to this study.

The efficacy of cannabis was validated through an investigation of the pathological features of *B. gonionotus* infected with *O. viverrini*. Findings from experiments revealed the presence of *O. viverrini* metacercariae embedded in the muscles of the fish, with a notable concentration in the head and eye muscles. Supplementing 2% cannabis leaves into the diet resulted in enhanced fibrous tissue formation and a greater presence of white blood cells surrounding metacercaria in fish compared to the control group that did not receive cannabis leaves. The group supplemented with cannabis leaves exhibited a prolonged increase in fibrous tissue formation and a higher concentration of white blood cells around the metacercaria. The fish infected with *O. viverrini* and fed 2.0% cannabis leaves for 21 days showed clear and dense fibrous tissue surrounding the metacercaria, which appeared to be shrinking. They exhibited characteristics of chronic inflammatory reactions, and the metacercaria were unable to grow into the infective stage. According to Berdyshev [41], cannabinoids affect both macrophages and T lymphocytes in the immune system, thereby influencing the host's defense against viral, bacterial, and protozoal infections. Moreover, phagocytosis may play an important role in the destruction of *O. viverrini* metacercariae, particularly through the activity of macrophages. Macrophages are crucial components of both the innate and adaptive immune systems. In innate immunity, they function by secreting acute-phase cytokines, eliminating pathogens via phagocytosis, and releasing inflammation mediators [42]. Cannabinoids, acting through the Cannabinoid Receptors CB1 and CB2, may modulate macrophage activity. These receptors are highly expressed in mouse and macrophages, microglial cells, and monocytes, regardless of cell type [42, 43]. Therefore, it is plausible that cannabinoid-mediated modulation of macrophages enhances their phagocytosis function, contributing to the destruction of metacercariae in fish. This experiment suggests that cannabinoids in cannabis leaves mixed in food formulas may stimulate white blood cells to surround and destroy macrophages, as well as to create thick fibrous tissue around the macrophages, preventing them from developing into an infective stage.

At the end of the study, an analysis was conducted on the tissue of fish that had been provided with a dietary supplement containing cannabis leaves to determine the presence of THC. The results revealed that THC was not detected in the fish tissue, suggesting that the cannabis leaves used as a supplement did not lead to the accumulation of THC in the fish tissue (Central Laboratory [Thailand] Co., Ltd.). Consequently, individuals can safely consume fish that is free from cannabis contamination, without experiencing any lingering effects on the nervous system.

## 5. Conclusions

This study concludes that supplementation of cannabis leaves at 2.0% in the feed can prevent infection by metacercariae of the liver fluke in silver barb fish on Days 14 and 21. The supplementation of cannabis leaves was effective in preventing infection, reducing infection intensity, decreasing the survival rate of metacercariae, and enhancing the immune response in fish. The metacercaria encysted within fibrous tissue was densely surrounded by white blood cells, leading to their destruction and inhibiting the growth of the liver fluke parasite. Consequently, consumers can safely consume fish devoid of liver fluke parasites and fish meat free from any residual contaminants.

## Figures and Tables

**Table 1:** The infection rate of *O. viverrini* metacercaria in infected fish fed diets supplemented with cannabis leaves at different levels.

**Feeding period (days)**	**Level of dietary supplementation of cannabis leaves in *B. gonionotus* (%)**				
	**0.0**	**0.5**	**1.0**	**1.5**	**2.0**
0	86.67 ± 5.77	93.33 ± 5.77	93.33 ± 5.77	90.00 ± 0.00	93.33 ± 5.77
7	93.33 ± 5.77^a^	80.00 ± 0.00^b^	76.67 ± 5.77^b^	73.33 ± 5.77^b^	63.33 ± 5.77^c^
14	93.33 ± 5.77^a^	53.33 ± 5.77^b^	46.67 ± 5.77^b^	3.33 ± 5.77^c^	0.00 ± 0.00^c^
21	93.33 ± 5.77^a^	63.33 ± 5.77^b^	46.67 ± 5.77^c^	13.33 ± 5.77^d^	0.00 ± 0.00^e^

*Note:* The different superscript letters (a–e) differ significantly (*p* < 0.05) in the same row.

**Table 2:** The intensity of *O. viverrini* metacercaria in infected fish fed diets supplemented with cannabis leaves at different levels.

**Feeding period (days)**	**Level of dietary supplementation of cannabis leaves in *B. gonionotus* (%)**				
	**0.0**	**0.5**	**1.0**	**1.5**	**2.0**
0	11.42 ± 0.33	10.88 ± 0.73	10.68 ± 0.16	10.77 ± 0.58	10.59 ± 0.42
7	11.13 ± 0.15^a^	9.54 ± 0.47^b^	8.25 ± 0.69^c^	4.15 ± 0.59^d^	0.74 ± 0.10^e^
14	10.37 ± 0.86^a^	4.90 ± 0.55^b^	3.25 ± 0.58^c^	0.67 ± 0.57^d^	0.00 ± 0.00^d^
21	11.13 ± 0.77^a^	6.65 ± 1.26^b^	6.55 ± 0.93^b^	2.44 ± 0.96^c^	0.00 ± 0.00^d^

*Note:* The different superscript letters (a–e) differ significantly (*p* < 0.05) in the same row.

**Table 3:** The survival rates of *O. viverrini* metacercaria in infected fish fed dietary cannabis leaf supplementation at different levels.

**Feeding period (days)**	**Levels of dietary supplementation of cannabis leaves in *B. gonionotus* (%)**				
	**0.0**	**0.5**	**1.0**	**1.5**	**2.0**
0	20.26 ± 0.30	20.26 ± 0.50	19.93 ± 0.92	19.40 ± 1.05	19.53 ± 0.11
7	20.20 ± 0.52^a^	12.00 ± 0.72^b^	9.53 ± 0.23^c^	3.33 ± 0.30^d^	0.46 ± 0.11^e^
14	19.93 ± 0.46^a^	3.33 ± 1.10^b^	1.46 ± 0.50^c^	0.13 ± 0.11^d^	0.00 ± 0.00^d^
21	19.86 ± 0.30^a^	3.80 ± 0.20^b^	1.73 ± 0.64^c^	0.26 ± 0.11^d^	0.00 ± 0.00^d^

*Note:* The different superscript letters (a–e) differ significantly (*p* < 0.05) in the same row.

## Data Availability

The data sets developed and analyzed were included in this study.

## Supporting Information

Supporting Information Additional supporting information can be found online in the Supporting Information section. The following supporting information can be downloaded at Figure 1: Superoxide dismutase activity (SOD).  Figure 2: Immunoglobulin M (IgM) levels and lysozyme activities.  Figure 3: Histology.  Figure 4: Histology.  Figure 5: Histology.  Figure 6: Histology.
