# Supplementary material for: Effects of Cannabis sativa L. Leaves on Opisthorchis viverrini Metacercariae in Infected Barbonymus gonionotus
Source: J Parasitol Res. 2025 Nov 11;2025:6233585. doi: 10.1155/japr/6233585 (PMC12626686; doi:10.1155/japr/6233585)
Supplement: Supporting Information — Additional supporting information can be found online in the Supporting Information section. The following supporting information can be downloaded at Figure 1: Superoxide dismutase activity (SOD). Figure 2: Immunoglobulin M (IgM) levels and lysozyme activities. Figure 3: Histology. Figure 4: Histology. Figure 5: Histology. Figure 6: Histology. [file 6233585.f1.docx]

**The data (raw files or meta data).**

TABLE 1: The fish were fed a diet mixed with cannabis leaves for 0 days, after which they were infected with *O. viverrini* cercariae (50 cercariae per fish).

| **Fish** | **Level of dietary supplementation of cannabis leaves in *B. gonionotus* (%)** | | | | | | | | | | | | | | |
| --- | --- | --- | --- | --- | --- | --- | --- | --- | --- | --- | --- | --- | --- | --- | --- |
|  | **0.0** | **0.0** | **0.0** | **0.5** | **0.5** | **0.5** | **1.0** | **1.0** | **1.0** | **1.5** | **1.5** | **1.5** | **2.0** | **2.0** | **2.0** |
| 1 | 1 | 1 | 1 | 1 | 1 | 1 | 1 | 1 | 1 | 1 | 1 | 1 | 1 | 1 | 1 |
| 2 | 1 | 1 | 1 | 1 | 1 | 1 | 1 | 1 | 1 | 1 | 1 | 1 | 1 | 1 | 1 |
| 3 | 1 | 1 | 0 | 1 | 1 | 1 | 1 | 1 | 1 | 1 | 1 | 1 | 1 | 1 | 1 |
| 4 | 1 | 0 | 1 | 0 | 1 | 1 | 0 | 1 | 1 | 1 | 0 | 1 | 1 | 0 | 1 |
| 5 | 1 | 1 | 1 | 1 | 1 | 1 | 1 | 1 | 1 | 1 | 1 | 1 | 1 | 1 | 1 |
| 6 | 1 | 1 | 0 | 1 | 1 | 1 | 1 | 1 | 1 | 1 | 1 | 0 | 1 | 1 | 1 |
| 7 | 0 | 1 | 1 | 1 | 0 | 1 | 1 | 1 | 1 | 0 | 1 | 1 | 0 | 1 | 1 |
| 8 | 1 | 1 | 1 | 1 | 1 | 1 | 1 | 1 | 0 | 1 | 1 | 1 | 1 | 1 | 1 |
| 9 | 1 | 1 | 1 | 1 | 1 | 1 | 1 | 1 | 1 | 1 | 1 | 1 | 1 | 1 | 1 |
| 10 | 1 | 1 | 1 | 1 | 1 | 1 | 1 | 1 | 1 | 1 | 1 | 1 | 1 | 1 | 1 |
| Total number of fish infected with liver fluke | 9 | 9 | 8 | 9 | 9 | 10 | 9 | 10 | 9 | 9 | 9 | 9 | 9 | 9 | 10 |
| Total number of fish infected with liver fluke / total fish x 100 | 90 | 90 | 80 | 90 | 90 | 100 | 90 | 100 | 90 | 90 | 90 | 90 | 90 | 90 | 100 |

* 0 = Not infected with liver fluke

*1 = Infected with liver fluke

TABLE 2: The fish were fed a diet mixed with cannabis leaves for 7 days, after which they were infected with *O. viverrini* cercariae (50 cercariae per fish).

| **Fish** | **Level of dietary supplementation of cannabis leaves in *B. gonionotus* (%)** | | | | | | | | | | | | | | |
| --- | --- | --- | --- | --- | --- | --- | --- | --- | --- | --- | --- | --- | --- | --- | --- |
|  | **0.0** | **0.0** | **0.0** | **0.5** | **0.5** | **0.5** | **1.0** | **1.0** | **1.0** | **1.5** | **1.5** | **1.5** | **2.0** | **2.0** | **2.0** |
| 1 | 1 | 1 | 1 | 1 | 1 | 1 | 1 | 1 | 1 | 1 | 1 | 0 | 1 | 1 | 1 |
| 2 | 1 | 1 | 1 | 1 | 0 | 1 | 1 | 1 | 0 | 1 | 1 | 1 | 0 | 0 | 1 |
| 3 | 1 | 1 | 0 | 1 | 1 | 0 | 1 | 0 | 1 | 1 | 1 | 1 | 0 | 1 | 0 |
| 4 | 1 | 1 | 1 | 0 | 1 | 1 | 0 | 1 | 1 | 0 | 0 | 1 | 1 | 1 | 0 |
| 5 | 1 | 1 | 1 | 1 | 1 | 1 | 1 | 1 | 1 | 1 | 1 | 1 | 1 | 0 | 1 |
| 6 | 1 | 1 | 1 | 1 | 1 | 0 | 1 | 0 | 1 | 1 | 0 | 0 | 0 | 1 | 1 |
| 7 | 0 | 1 | 1 | 0 | 0 | 1 | 0 | 1 | 1 | 0 | 1 | 1 | 1 | 1 | 0 |
| 8 | 1 | 1 | 1 | 1 | 1 | 1 | 1 | 0 | 0 | 1 | 0 | 1 | 1 | 1 | 1 |
| 9 | 1 | 1 | 1 | 1 | 1 | 1 | 1 | 1 | 1 | 1 | 1 | 0 | 1 | 0 | 1 |
| 10 | 1 | 1 | 1 | 1 | 1 | 1 | 1 | 1 | 1 | 1 | 1 | 1 | 0 | 1 | 0 |
| Total number of fish infected with liver fluke | 9 | 10 | 9 | 8 | 8 | 8 | 8 | 7 | 8 | 8 | 7 | 7 | 6 | 7 | 6 |
| Total number of fish infected with liver fluke / total fish x 100 | 90 | 100 | 90 | 80 | 80 | 80 | 80 | 70 | 80 | 80 | 70 | 70 | 60 | 70 | 60 |

* 0 = Not infected with liver fluke

*1 = Infected with liver fluke

TABLE 3: The fish were fed a diet mixed with cannabis leaves for 14 days, after which they were infected with *O. viverrini* cercariae (50 cercariae per fish).

| **Fish** | **Level of dietary supplementation of cannabis leaves in *B. gonionotus* (%)** | | | | | | | | | | | | | | |
| --- | --- | --- | --- | --- | --- | --- | --- | --- | --- | --- | --- | --- | --- | --- | --- |
|  | **0.0** | **0.0** | **0.0** | **0.5** | **0.5** | **0.5** | **1.0** | **1.0** | **1.0** | **1.5** | **1.5** | **1.5** | **2.0** | **2.0** | **2.0** |
| 1 | 1 | 1 | 1 | 1 | 0 | 1 | 1 | 0 | 0 | 0 | 0 | 0 | 0 | 0 | 0 |
| 2 | 1 | 1 | 1 | 0 | 0 | 1 | 0 | 1 | 1 | 0 | 0 | 0 | 0 | 0 | 0 |
| 3 | 1 | 1 | 1 | 1 | 1 | 1 | 1 | 1 | 0 | 0 | 0 | 0 | 0 | 0 | 0 |
| 4 | 1 | 1 | 1 | 0 | 1 | 0 | 1 | 1 | 1 | 0 | 0 | 1 | 0 | 0 | 0 |
| 5 | 1 | 0 | 1 | 0 | 1 | 0 | 0 | 0 | 0 | 0 | 0 | 0 | 0 | 0 | 0 |
| 6 | 1 | 1 | 1 | 1 | 0 | 1 | 0 | 0 | 0 | 0 | 0 | 0 | 0 | 0 | 0 |
| 7 | 1 | 1 | 1 | 1 | 1 | 0 | 1 | 1 | 0 | 0 | 0 | 0 | 0 | 0 | 0 |
| 8 | 1 | 1 | 1 | 1 | 0 | 0 | 0 | 1 | 1 | 0 | 0 | 0 | 0 | 0 | 0 |
| 9 | 0 | 1 | 1 | 1 | 1 | 0 | 0 | 0 | 0 | 0 | 0 | 0 | 0 | 0 | 0 |
| 10 | 1 | 1 | 1 | 0 | 0 | 1 | 1 | 0 | 1 | 0 | 0 | 0 | 0 | 0 | 0 |
| Total number of fish infected with liver fluke | 9 | 9 | 10 | 6 | 5 | 5 | 5 | 5 | 4 | 0 | 0 | 1 | 0 | 0 | 0 |
| Total number of fish infected with liver fluke / total fish x 100 | 90 | 90 | 100 | 60 | 50 | 50 | 50 | 50 | 40 | 0 | 0 | 10 | 0 | 0 | 0 |

* 0 = Not infected with liver fluke

*1 = Infected with liver fluke

TABLE 4: The fish were fed a diet mixed with cannabis leaves for 21 days, after which they were infected with *O. viverrini* cercariae (50 cercariae per fish).

| **Fish** | **Level of dietary supplementation of cannabis leaves in *B. gonionotus* (%)** | | | | | | | | | | | | | | |
| --- | --- | --- | --- | --- | --- | --- | --- | --- | --- | --- | --- | --- | --- | --- | --- |
|  | **0.0** | **0.0** | **0.0** | **0.5** | **0.5** | **0.5** | **1.0** | **1.0** | **1.0** | **1.5** | **1.5** | **1.5** | **2.0** | **2.0** | **2.0** |
| 1 | 1 | 1 | 1 | 1 | 0 | 1 | 1 | 1 | 1 | 1 | 0 | 1 | 0 | 0 | 0 |
| 2 | 1 | 1 | 0 | 1 | 0 | 1 | 1 | 0 | 1 | 0 | 1 | 1 | 0 | 0 | 0 |
| 3 | 1 | 1 | 1 | 1 | 1 | 0 | 1 | 0 | 0 | 1 | 1 | 1 | 0 | 0 | 0 |
| 4 | 1 | 1 | 1 | 0 | 1 | 1 | 0 | 0 | 0 | 0 | 1 | 1 | 0 | 0 | 0 |
| 5 | 1 | 1 | 1 | 0 | 1 | 1 | 1 | 1 | 1 | 1 | 1 | 0 | 0 | 0 | 0 |
| 6 | 0 | 1 | 1 | 1 | 0 | 0 | 1 | 1 | 1 | 1 | 1 | 1 | 0 | 0 | 0 |
| 7 | 1 | 1 | 1 | 1 | 1 | 1 | 0 | 1 | 0 | 1 | 1 | 1 | 0 | 0 | 0 |
| 8 | 1 | 1 | 1 | 0 | 1 | 0 | 0 | 0 | 1 | 1 | 1 | 1 | 0 | 0 | 0 |
| 9 | 1 | 1 | 1 | 1 | 0 | 0 | 0 | 0 | 0 | 1 | 1 | 1 | 0 | 0 | 0 |
| 10 | 1 | 1 | 1 | 1 | 1 | 1 | 0 | 0 | 0 | 1 | 1 | 1 | 0 | 0 | 0 |
| Total number of fish infected with liver fluke | 9 | 10 | 9 | 7 | 6 | 6 | 5 | 4 | 5 | 2 | 1 | 1 | 0 | 0 | 0 |
| Total number of fish infected with liver fluke / total fish x 100 | 90 | 100 | 90 | 70 | 60 | 60 | 50 | 40 | 50 | 20 | 10 | 10 | 0 | 0 | 0 |

* 0 = Not infected with liver fluke

*1 = Infected with liver fluke

TABLE 5: Data statistical analysis. (The infection rate)

| **Level of dietary supplementation of cannabis leaves in *B. gonionotus* (%)** | **replications** | **Feeding period (Days)** | | | |
| --- | --- | --- | --- | --- | --- |
|  |  | **0 day** | **7 days** | **14 days** | **21 days** |
| 0.0% | 1 | 90.00 | 90.00 | 90.00 | 90.00 |
|  | 1 | 90.00 | 100.00 | 90.00 | 100.00 |
|  | 1 | 80.00 | 90.00 | 100.00 | 90.00 |
| 0.5% | 2 | 90.00 | 80.00 | 60.00 | 70.00 |
|  | 2 | 90.00 | 80.00 | 50.00 | 60.00 |
|  | 2 | 100.00 | 80.00 | 50.00 | 60.00 |
| 1.0% | 3 | 90.00 | 80.00 | 50.00 | 50.00 |
|  | 3 | 100.00 | 70.00 | 50.00 | 40.00 |
|  | 3 | 90.00 | 80.00 | 40.00 | 50.00 |
| 1.5% | 4 | 90.00 | 80.00 | 0.00 | 20.00 |
|  | 4 | 90.00 | 70.00 | 0.00 | 10.00 |
|  | 4 | 90.00 | 70.00 | 10.00 | 10.00 |
| 2.0% | 5 | 90.00 | 60.00 | 0.00 | 0.00 |
|  | 5 | 90.00 | 70.00 | 0.00 | 0.00 |
|  | 5 | 100.00 | 60.00 | 0.00 | 0.00 |
